# Encystment Induces Down-Regulation of an Acetyltransferase-Like Gene in *Acanthamoeba castellanii*

**DOI:** 10.3390/pathogens9050321

**Published:** 2020-04-26

**Authors:** Steven Rolland, Luce Mengue, Cyril Noël, Stéphanie Crapart, Anne Mercier, Willy Aucher, Yann Héchard, Ascel Samba-Louaka

**Affiliations:** 1 UMR CNRS 7267 Ecologie et Biologie des Interactions, Université de Poitiers, Pôle Biologie-Santé, Bât. B36/B37, 1 rue Georges Bonnet, TSA 51106 86073 Poitiers CEDEX 9, France; steven.rolland1@laposte.net (S.R.); lucelaetitia@gmail.com (L.M.); stephanie.crapart@univ-poitiers.fr (S.C.); anne.mercier@univ-poitiers.fr (A.M.); willy.aucher@univ-poitiers.fr (W.A.); yann.hechard@univ-poitiers.fr (Y.H.); 2 IFREMER-IRSI-Service de Bioinformatique (SeBiMER), Centre Bretagne, 1625 Route de Sainte-Anne, 29280 Plouzane, France; cyril.noel@ifremer.fr

**Keywords:** *Acanthamoeba castellanii*, encystment, N-acetyltransferase, lateral gene transfer, free-living amoebae

## Abstract

*Acanthamoeba castellanii* is a ubiquitous free-living amoeba. Pathogenic strains are causative agents of *Acanthamoeba* keratitis and granulomatous amoebic encephalitis. In response to adverse conditions, *A. castellanii* differentiate into cysts, which are metabolically inactive and resistant cells. This process, also named encystment, involves biochemical and genetic modifications that remain largely unknown. This study characterizes the role of the *ACA1_384820* *Acanthamoeba* gene during encystment. This gene encodes a putative N-acetyltransferase, belonging to the Gcn5-related N-acetyltransferase (GNAT) family. We showed that expression of the *ACA1_384820* gene was down-regulated as early as two hours after induction of encystment in *A. castellanii*. Interestingly, overexpression of the *ACA1_384820* gene affects formation of cysts. Unexpectedly, the search of homologs of *ACA1_384820* in the Eukaryota gene datasets failed, except for some species in the *Acanthamoeba* genus. Bioinformatics analysis suggested a possible lateral acquisition of this gene from prokaryotic cells. This study enabled us to describe a new *Acanthamoeba* gene that is down-regulated during encystment.

## 1. Introduction

*Acanthamoeba* spp. are free-living amoebae, commonly found in diverse natural environments such as soil, water and air, and also in artificial facilities including tap water systems, cooling towers, sewage and air conditioning systems [1,2]. Pathogenic strains of *Acanthamoeba* spp. are causative agents of a rare fatal infection of the central nervous system, called granulomatous amoebic encephalitis, and of *Acanthamoeba* keratitis (AK), a progressive eye disease [3,4]. AK is generally associated with contact lens wearers and remains an elusive problem in spite of advances in antimicrobial chemotherapy and eye care [5]. 

*Acanthamoeba’*s life cycle consists of two stages: a metabolically active trophozoite and an inactive dormant cyst [3]. The formation of cysts, also called encystment, is a reversible process that is induced by harsh conditions, such as lack of nutrients, or change in osmotic pressure or pH [6,7]. The cyst represents a resistant and resting form that protects the amoeba against adverse conditions such as heat, freezing and chemicals, and enables it to persist for many years [8,9]. 

*Acanthamoeba* feed on bacteria but some of the bacteria are able to resist digestion by the amoeba. *Acanthamoeba* thereby act as ‘Trojan horses;’ of the bacterial world and are considered as a training ground for pathogenic bacteria insofar as the passage through amoebae may be associated with an increase of bacterial virulence [10]. In their cyst form, *Acanthamoeba* are able to keep and protect intracellular bacteria from biocides, leading to recurrent infections [11,12]. Interestingly, some intracellular bacteria have been reported to modulate amoeba metabolic functions such as encystment. *Francisella tularensis* thereby induces the encystment process, unlike *Parachlamydiae acanthamoebae*, which inhibits it [13,14]. Analysis of the *Acanthamoeba* genomes suggests genetic exchanges with intracellular microorganisms [15,16]. The genome of *Acanthamoeba castellanii* contains 2.9% of genes possibly acquired through lateral gene transfer (LGT). Among them, several are annotated as ‘acetyltransferase, Gcn5-related N-acetyltransferase (GNAT) superfamily protein’. GNATs are a very large family of enzymes, comprising more than 10,000 members, that are identified in all kingdoms of life [17]. GNATs catalyze the transfer of an acetyl group from acetyl-CoA to the primary amine of a large range of substrates, from small molecules to macromolecules [18]. LGT-derived genes are differentially expressed in *A. castellanii* following the growth phase, under agitation, hypoxia or upon bacterial infections, suggesting their involvement in *Acanthamoeba* physiology [16]. Nevertheless, the contribution of these LGT-derived genes to encystment remains under-investigated. 

Regarding their importance in cell physiology, we have investigated the role of putative GNATs in the encystment of *A. castellanii*. Analysis of the expression of these genes was performed from 2 to 24 h after incubation of *A. castellanii* within an encystment medium. A plasmid vector was constructed to overexpress the acetyltransferase-like *ACA1_384820* based on an effective and stable transfection approach. 

## 2. Results

### 2.1. The N-Acetyltransferase-Like mRNA Levels Are Down-Regulated during Encystment 

**Figure 1:**
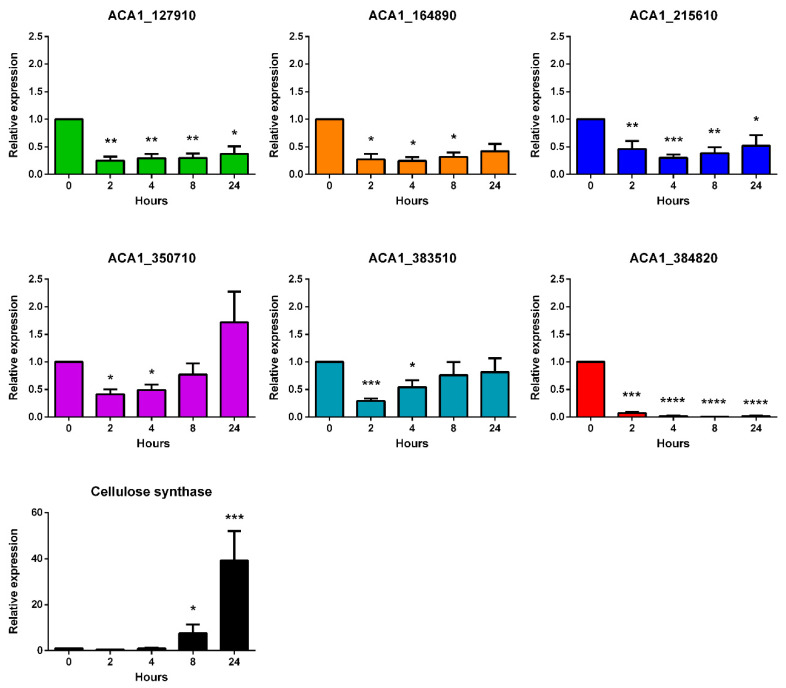
The expression of the *A. castellanii* N-acetyltransferase-like genes is down-regulated during encystment. The relative expression of *ACA1_127910*, *ACA1_164890*, *ACA1_215610*, *ACA1_350710*, *ACA1_164890*, *ACA1_384820* and cellulose synthase genes was assessed by RT-qPCR 0, 2, 4, 8 and 24 h after induction of the encystment. Results represent average values of three independent experiments, and error bars represent the standard error of the mean (± SEM). Statistical analysis was performed using the ordinary one-way ANOVA followed by Dunnett’s post hoc test (* *p* <  0.05, ** *p* < 0.01, *** *p* < 0.001, **** *p* < 0.0001) from ΔCt values. At each time point, means were compared to the ‘0 h’ condition.

The genome of *A. castellanii* contains several N-acetyltransferase-like genes such as *ACA1_127910*, *ACA1_164890*, *ACA1_350710*, *ACA1_383510*, *ACA1_215610* and *ACA1_384820* [16]. In order to study their expression during encystment of *A. castellanii*, real-time qPCR (RT-qPCR) was conducted at 0, 2, 4, 8 and 24 h after induction of the encystment. Expression of the cellulose synthase gene was also analyzed as a marker of encystment [19]. As can be seen in Figure 1, the mRNA levels of N-acetyltransferase-like genes decreased as soon as 2 h after the induction of encystment. This decrease persisted up to 24 h for the *ACA1_127910*, *ACA1_164890*, *ACA1_215610* and *ACA1_384820* genes. However, the down-regulation of *ACA1_350710* and *ACA1_383510* was transient. In contrast and as expected, cellulose synthase was over-expressed at 24 h after the induction of encystment. These results suggest that the incubation of *A. castellanii* into the encystment medium induces down-regulation, at least transiently, of the N-acetyltransferase-like genes. Down-regulation of the ACA1_384820 gene was more pronounced compared to the other N-acetyltransferase-like genes studied. We selected this gene for the subsequent experiments. 

### 2.2. The ACA1_384820 Gene Encodes a Putative N-Acetyltransferase-Like Protein That Presents Homologies with Prokaryotic Sequences 

A bioinformatics analysis was performed to characterize the putative function of the *ACA1_384820* gene. The *ACA1_384820* gene encodes a putative protein of 345 amino acids [20]. The coding sequence of ACA1_384820 was amplified from the cDNA extracted from the *A. castellanii* str. Neff (ATCC 30010) and the nucleotide sequence obtained missed 21 nucleotides corresponding to 7 amino acids (Supplementary Data 1 and 2). This deletion did not change either the open reading frame or the putative active domain of the predicted protein. 

The identification of specific domains with InterPro showed the presence of a Gcn5-related N-acetyltransferase (GNAT) domain in the C-terminal region. No additional domain was predicted. Accordingly, the ACA1_384820 protein could be a putative N-acetyltransferase, a family of proteins involved in the transfer of the acetyl group from acetyl-CoA to a substrate. 

In order to find homologs of the ACA1_384820 protein, a Basic Local Alignment Search Tool (BLAST) analysis was performed against the National Center for Biotechnology Information (NCBI) non-redundant protein sequence database. Excluding *A. castellanii*, the best hits (e-value threshold < 1e-10) were affiliated to GNAT-family N-acetyltransferases belonging to Bacteria and Archaea with around 30% of identity (Supplementary Data 3). No homology was found within Eukaryota. In addition, in-depth analysis for the presence of *ACA1_384820* within all available amoeba genomes (annotated, non-annotated and not present in the NCBI database) was conducted on 27 genomes from *Acanthamoeba* spp., *Dictyostelium* spp., *Entamoeba* spp. and *Naegleria* spp. Some homologous genes were identified in *Acanthamoeba* (*pearcei*, *quina*, *lugdunensis*, *polyphaga*, *rhysodes*, *palestinensis* and *mauritaniensis*) (Table 1, Supplementary Data 4). No homologous genes were found in the genomes of *Naegleria* (*fowleri, gruberi, lovaniensis*), *Entamoeba* (*histolytica, dispar, nuttalli, invandens, moshkovskii*), *Dictyostelium* (*discoideum, citrinum, purpureum, intermedium, firmibasis*) and some *Acanthamoeba* (*lenticulata, astronyxis, comandoni, divionensis, royreba, healyi, culbertsoni*). According to these results, the *ACA1_384820* gene seems very specific to *Acanthamoeba* spp. 

### 2.3. Overexpression of the ACA1_384820 Gene Affects the Formation of Cysts

**Figure 2:**
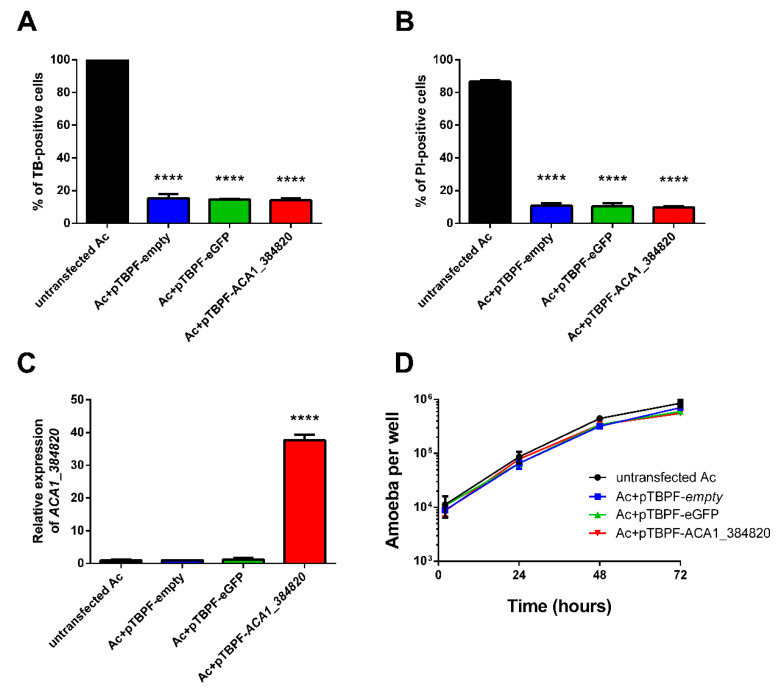
Overexpression of the *ACA1_384820* gene does not strongly affect the growth of *A. castellanii*. Evaluation of cell viability using (**A**) Trypan blue (TB) or (**B**) propidium iodide (PI) on the transfected cells, five days after the increase of G418 antibiotic concentration. TB- or PI-positive cells were considered as non-viable. (**C**) Relative expression of the *ACA1_384820* gene in *A. castellanii* transfected or not with plasmids. (**D**) Evaluation of the impact of plasmid transfection on the growth of *A. castellanii* in Peptone Yeast Glucose (PYG) medium at 30 ℃. Results are the average of three independent experiments, and error bars represent the standard error of the mean (± SEM). Statistical analysis was performed using the ordinary one-way ANOVA followed by Dunnett’s post hoc test (* *p* < 0.05, ** *p* < 0.01, *** *p* < 0.001, **** *p* < 0.0001) with comparison to the condition ‘untransfected Ac’.

To better characterize the function of the *ACA1_384820* gene, we examined whether its overexpression could affect encystment. A plasmid with the *ACA1_384820* coding sequence (pTBPF- *ACA1_384820*) was constructed. In this plasmid, the *ACA1_384820* coding sequence was under the promoter of the TATA-binding protein promoter binding factor (TPBF) for constitutive expression [21]. Indeed, the mRNA of TPBF is stable during encystment [22]. Plasmids expressing eGFP (TBPF-eGFP) or nothing (TBPF-empty) were used as controls. Plasmids were transfected into *A. castellanii,* followed by selective treatment with Geneticin (G418). Cell death was assessed using Trypan blue and propidium iodide. Twenty percent of transfected cells were positive for both Trypan blue and propidium iodide, while all non-transfected cells were dead (Figure 2A-B). To determine whether *ACA1_384820* was overexpressed within the transfected cells, we analyzed its expression by RT-qPCR. In cells transfected with pTBPF-*ACA1_384820*, an increased *ACA1_384820* mRNA level was observed (Figure 2C). In contrast, cells transfected with pTBPF-empty or pTBPF-eGFP displayed a similar level compared to untransfected cells (Figure 2C). 

In order to assess whether the overexpression of the *ACA1_384820* gene altered the growth of amoebae, transfected and control cells were counted every 24 h for 72 h. *A. castellanii* growth was not affected by overexpression of the *ACA1_384820* gene compared to untransfected cells, or those which were transfected with the two control plasmids pTBPF-eGFP and pTBPF-empty (Figure 2D). Altogether, these results show that overexpression of the *ACA1_384820* coding sequence did not disturb the growth of *A. castellanii*.

**Figure 3:**
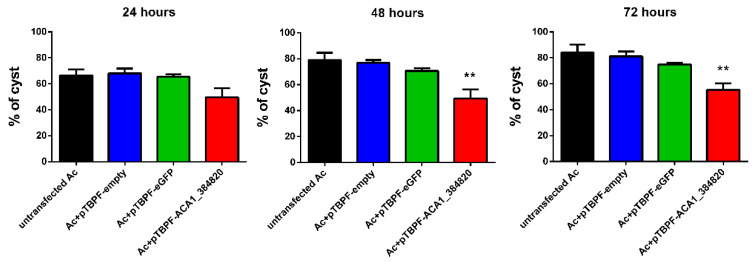
Overexpression of ACA1_384820 coding sequence affects formation of cysts. Encystment of *A. castellanii* was induced in encystment medium. At 24, 48 and 72 h, Calcofluor White was added into the wells and the percentage of Calcofluor-positive cells was estimated in the total population. Results are averages of three independent experiments, and error bars represent the standard error of the mean (± SEM). Statistical analysis was performed using ordinary one-way ANOVA followed by Dunnett’s post hoc test (** *p* < 0.01) in comparison to the condition ‘untransfected Ac’.

Finally, we tested the influence of *ACA1_384820* overexpression on encystment of *A. castellanii*. We incubated transfected amoebae within the encystment medium and evaluated the percentage of cysts using the Calcofluor White stain at different time points (24, 48 and 72 h). Twenty-four hours after the induction of encystment, we observed a decrease in the percentage of cysts. This effect was more pronounced at 48 and 72 h (Figure 3). In conclusion, *ACA1_384820* overexpression affects formation of cysts. 

## 3. Discussion

The goal of this study was to find new proteins down-regulated during encystment of *Acanthamoeba*. The *ACA1_384820* gene encodes for a putative acetyltransferase, according to the domain prediction showing the presence of a GNAT domain. GNATs are a very large family of enzymes, with more than 10,000 members, identified in all kingdoms of life [17]. GNATs catalyze the transfer of an acetyl group from acetyl-CoA to the primary amine of a large range of substrates, ranging from small molecules to macromolecules. More globally, among post-translational modifications (PTM), acetylation is able to modulate the function of target molecules implicated in numerous cellular processes, ranging from antibiotic resistance to gene regulation by histone acetylation for example [18]. Some N-acetyltransferases have been described as being involved in encystment of other eukaryotes. In *Giardia intestinalis*, the cyst-specific carbohydrate component (67%) of the cyst wall is a unique homopolymer composed of N-acetylgalactosamine (GalNAc). Its precursor, UDP-GalNAc is synthesized by a pathway of five inducible enzymes, including one glucosamine 6-phosphate N-acetyltransferase (EC 2.3.1.4, GNA) [23,24]. In *Toxoplasma gondii*, it has been shown that the knock-out of TgGCN5-A, a lysine acetyltransferase with a histone acetyltransferase activity, prevented up-regulation of 74% of stress response genes that are normally induced during alkaline stress-mediated encystment. Complementation of the TgGCN5-A knock-out restored this expression and the capacity to resist alkaline stress, underlining the role of a N-acetyltransferase in encystment [25]. These two examples demonstrate the involvement of GNAT members in encystment. 

We found that expression of the putative *Acanthamoeba* GNATs was down-regulated during encystment. This decrease was transient or persisted depending on GNATs, suggesting an action at different times with different targets and outcomes. In the case of the *ACA1_384820* gene, overexpression impaired formation of mature cysts. This protein might modulate molecules that negatively affect encystment in *A. castellanii*. Ongoing work consists of determining the molecular targets and activities of the different GNATs. Further studies are needed to analyze the precise role of each acetyltransferases and how they are coordinated during encystment. Indeed, if several N-acetyltransferases are needed to prevent encystment, this could explain why overexpression of the *ACA1_384820* gene does not completely block formation of cysts.

The analysis of amoeba genomes showed that the *ACA1_384820* gene is conserved in some species of *Acanthamoeba*, but absent in *Dictyostelium* spp., *Entamoeba* spp. and *Naegleria* spp. The search for the protein sequence within other organisms using the BLAST tool have shown some identities with proteins mainly found in prokaryotes and belonging to the phyla of Chlorobacteria, Cyanobacteria and Firmicutes. In the environment, amoebae are in contact with numerous bacteria on which they graze [26]. Due to putative gene exchanges between amoebae and intracellular bacteria, it was not surprising to find an *Acanthamoeba* gene of prokaryotic origin. The study of the *Acanthamoeba* genome shows the presence of more than 450 genes, which corresponds to 2.9% of the genome, predicted to have arisen through lateral gene transfer [16]. Among these genes, we have *ACA1_384820* and at least five genes that have been annotated as ‘acetyltransferase, GNAT superfamily protein’. All these genes are down-regulated during encystment. These data suggest that amoebae could have acquired bacterial genes that are involved in encystment but further analysis is required to confirm the hypothesis.

In conclusion, we describe a new *ACA1_384820* gene of which the expression is down-regulated during encystment of *A. castellanii.* Overexpression of *ACA1_384820* affects formation of cysts. This protein encodes a putative N-acetyltransferase-like protein possibly acquired by lateral gene transfer from prokaryotes. Further studies are needed to determine the activity of this protein and its specific role in encystment of *A. castellanii*. 

## 4. Materials and Methods

### 4.1. Amoeba Strains and Cultural Conditions

*A. castellanii* ATCC 30010 was grown at 30 °C without shaking, in a culture flask containing Peptone Yeast Glucose (PYG) medium (2% proteose peptone, 0.1% yeast extract, 0.1 M glucose, 4 mM MgSO_4_, 0.4 mM CaCl_2_, 0.1% sodium citrate dihydrate, 0.05 mM Fe(NH_4_)_2_(SO_4_)_2_ 6H_2_O, 2.5 mM NaH_2_PO_3_, 2.5 mM K_2_HPO_3_, pH 6.5). For the transfected cells, G418 (Geneticin) was used at 50 µg/mL to maintain plasmids.

For the growth assay, *A. castellanii* trophozoites were seeded onto 24-well plates at a density of 5 × 10^4^ cells per well in 1 mL of Page’s Amoeba Saline solution (PAS) (4 mM MgSO_4_, 0.4 mM CaCl_2_, 0.1% sodium citrate dehydrate, 0.05 mM Fe(NH_4_)_2_(SO_4_)_2_ 6H_2_O, 2.5 mM NaH_2_PO_3_, 2.5 mM K_2_HPO_3_, pH 6.5) and incubated at 30 ℃ for 1 h for cell adhesion. Then, PAS buffer was replaced by PYG growth medium (corresponding to time 0) and incubated at 30 ℃. Cells were harvested at 2, 24, 48 and 72 h and counted using plastic counting slides FastRead 102^®^ (Biosigma). All samples were counted three times and in three independent experiments.

### 4.2. Encystment Assay 

*A. castellanii* transfected and non-transfected trophozoites were seeded onto 24-well plates at a density of 5 × 10^4^ cells per well in PAS buffer and incubated at 30 °C for 1 h for cell adhesion. PAS was then replaced by an encystment medium (0.1 M KCl, 8 mM MgSO_4_, 0.4 mM CaCl_2_, 1 mM NaHCO_3_ and 20 mM 2-amino-2-methyl-1,3-propanediol, pH 8.8) and incubated at 30 °C (corresponding to time 0) up to 72 h. At 24, 48 and 72 h, Calcofluor White Reagent (Becton Dickinson), a dye that binds to cellulose, was incubated with live *A. castellanii* on a glass slide for 2 min at room temperature [27]. The cysts were observed by fluorescence microscopy (Olympus IX51). More than 800 cells were counted per condition and per experiment. This experiment was done in three independent replicates. 

### 4.3. Plasmid Constructions and Cloning

The *ACA1_384820* coding sequence from *A. castellanii* was amplified by PCR from total cDNA with flanking NdeI and XhoI restriction sites, using the primers ACA1_384820_Fwd_NdeI and ACA1_384820_Rev_XhoI (Table 2). PCR fragments were cloned into the NdeI/XhoI sites of the expression plasmid pTBPF-eGFP [21]. For the pTBPF-empty, pTBPF-eGFP was digested by NdeI and XhoI (NEB), and the sticky 5’-overhangings ends of the vector were filled using DNA Polymerase I Large (Klenow) Fragment (Promega) and ligated (T4 DNA Ligase, Promega) following the manufacturer’s recommendations. 

All plasmid constructs were transferred in chemically-competent *Escherichia coli* DH5α and validated by Sanger sequencing. DNA sequencing was completed with the ABI Prism BigDye™ terminator v3.1 sequencing kit (Applied Biosystems) and then analyzed using an automatic ABI Prism 3730 genetic analyzer (Applied Biosystems).

### 4.4. Transfection of Cells 

*A. castellanii* trophozoites were seeded into 24-well plates at a density of 1.25 × 10^5^ cells per well in 125 µL of the encystment medium. The Viafect™ transfection reagent (Promega) was used to transfect plasmid into *A. castellanii*. Aliquots (1 µg) of plasmid vectors were incubated with the transfection reagent at a ratio of 5:1 (transfection reagent (μL)/plasmid (μg)) in 150 µL of the encystment medium and incubated for 15 min at room temperature. Transfection medium was added directly to the cells and incubated for 3 h at 30 °C without shaking, before the addition of 750 µL of PYG medium. After a 24 h incubation, the well contents were transferred into culture flasks containing PYG medium supplemented with 20 µg/mL of G418 (Sigma) for two weeks. The concentration of G418 was then increased to 50 µg/mL to select the transfected population.

Five days after the increase of G418 concentration, cell viability was tested using Trypan blue and propidium iodide. For the Trypan blue experiments, cells were diluted in 2× Trypan blue (final concentration of 0.2%) and counted in triplicate for each condition using plastic counting slides FastRead 102^®^ (Biosigma). For the propidium iodide experiments, cells were incubated with propidium iodide (10 µg/mL) and analyzed by flow cytometry (Cytoflex, Beckman Coulter). This experiment was done in three independent replicates.

### 4.5. Reverse Transcription-Quantitative PCR (RT-qPCR)

Total RNAs was extracted using the RNeasy Mini Kit (Qiagen). For samples incubated in encysting medium for at least 24 h, RNA extraction was preceded by physical lysis by bead-beating in tubes containing 500 mg of small diameter glass beads (100 μm) (Sigma) using Fastprep apparatus for 30 s (speed 5 m/s). The RNA samples were treated with RNase-free DNase I (TURBO DNA-*free*™ kit, Invitrogen) and reverse transcribed with the GoScript™ Reverse Transcriptase kit (Promega) according to the manufacturer’s recommendations. The reverse transcription products were used to carry out real-time quantitative PCR. All primer sequences are shown in Table 2. Reverse Transcription-Quantitative PCR (RT-qPCR) was performed using the LightCycler^®^ FastStart DNA Master plus SYBR Green I (Roche Applied Science). Reactions were prepared in a total volume of 10 µL containing 5 µL of 2× SYBR mix, 2 µL of H_2_O, 2 µL of diluted cDNA template and 0.5 µL of 10 µM primers.

The reactions were performed under the following conditions: an initial denaturation step of 95 °C for 5 min, followed by a three-step thermal cycling profile comprising denaturation at 95 °C for 10 s, primer annealing at 60 ℃ for 10 s and extension at 72 ℃ for 10 s. This procedure was conducted for 45 cycles. To verify the specificity of the amplicon for each primer pair, a melting curve analysis was performed ranging from 65 to 95 ℃. 

The relative quantification method (2^-ΔΔCt^) was used to evaluate quantitative variation between replicates [29]. The relative expression of the six N-acetytransferase-like (*ACA1_127910*, *ACA1_164890*, *ACA1_215610*, *ACA1_350710*, *ACA1_164890*, *ACA1_384820*) and cellulose synthase (*ACA1_349650*) genes were normalized towards *tbpf* gene (NCBI accession: L46867.1). The overexpression of ACA1_384820 in transfected amoebae was also confirmed using the 18S rRNA gene as reference gene.

### 4.6. Bioinformatics Analysis of the ACA1_384820 Gene 

Predictions of potential domains present on the protein were performed using online tool InterPro [30,31]. The search for putative homologous genes in other organisms were performed using the online bioinformatics tool BLASTp [32]. To generate alignment (Supplementary Data 1 and 2), the sequences were analyzed by Multalin [33,34]. For amino acid sequences, the nucleotide sequences were translated using ExPasy tool [35]. 

### 4.7. Statistical Analysis

All results are averages of three independent experiments, and error bars represent the standard error of the mean (± SEM). Statistical analysis was performed using the ordinary one-way ANOVA followed by Dunnett’s post hoc test (GraphPad Prism 6). For RT-qPCR experiments, statistical analyses were performed on ΔCt values. Differences were considered statistically significant when *p* values were < 0.05 (* *p* < 0.05; ** *p* < 0.01; *** *p* < 0.001; **** *p* < 0.0001).

## Figures and Tables

**Table 1:** The *ACA1_384820* homologs in amoeba genomes.

Query_ID	Subject_ID	Organisms	% Identity	Length	Start (query)	End (query)	Start (subject)	End (subject)	e-Value	Bitscore
*ACA1_384820*	NW_004457598.1	*Acanthamoeba castellanii*	100.00	1228	1	1228	109079	110306	0.0	2268
	CDFJ01209063.1	*Acanthamoeba pearcei*	88.53	1247	5	1228	1729	497	0.0	1476
	CDFJ01209064.1		88.42	1244	8	1228	1726	497	0.0	1465
	CDFN01057746.1	*Acanthamoeba quina*	88.04	1237	5	1221	2781	3987	0.0	1419
	CDFB01039259.1	*Acanthamoeba lugdunensis*	88.04	1221	18	1221	1291	2478	0.0	1400
	LQHA01001417.1	*Acanthamoeba polyphaga*	87.66	1224	23	1221	1807	599	0.0	1387
	CDFC01048720.1	*Acanthamoeba rhysodes*	86.14	1255	20	1218	404	1633	0.0	1279
	CDFC01052420.1		87.56	595	635	1218	1485	2073	0.0	673
	CDFC01052420.1		85.54	415	238	632	992	1404	1.98e-113	414
	CDFC01052420.1		88.54	192	4	195	722	913	5.94e-59	233
	CDFD01054069.1	*Acanthamoeba palestinensis*	79.70	1266	5	1228	50106	51339	0.0	846
	CDFE01061388.1	*Acanthamoeba mauritaniensis*	89.29	588	635	1218	2725	3307	0.0	728
	CDFE01061388.1		83.36	667	12	631	2021	2676	1.85e-158	564
	CDFE01051786.1		89.12	588	635	1218	1082	500	0.0	723
	CDFE01051786.1		85.78	647	18	631	1746	1110	0.0	645

**Table 2:** Primers used for RT-qPCR and plasmid constructions.

Name of Primer	Sequence 5’ -> 3’	Use	Source
**qACA1_127910_Fwd**	GCGCATCTTCTTCATCGAGG	RT-qPCR	This study
**qACA1_127910_Rev**	CTTGTCGTTCGAACCCTTGG	RT-qPCR	This study
**qACA1_164890_Fwd**	TTCTTCATCGAGGAGGAGGC	RT-qPCR	This study
**qACA1_164890_Rev**	CGTCCAGTTTGAGTAGTGCG	RT-qPCR	This study
**qACA1_350710_Fwd**	CATGCTCAACGACATCACCC	RT-qPCR	This study
**qACA1_350710_Rev**	GTACTCCACCACTTCCACCT	RT-qPCR	This study
**qACA1_383510_Fwd**	GAGAATGGCGGCATGAATCC	RT-qPCR	This study
**qACA1_383510_Rev**	GCGCTCTTTCGTGATGTCAA	RT-qPCR	This study
**qACA1_215610_Fwd**	GAAGATGGGGTTCGTGCAGA	RT-qPCR	This study
**qACA1_215610_Rev**	TCGGTTTCTGGAAGGAGAGG	RT-qPCR	This study
**qACA1_384820_Fwd**	TTTCGCCCAGAAGCCCAGAG	RT-qPCR	This study
**qACA1_384820_Rev**	TCGTTCAGGTGGCGTAGCAG	RT-qPCR	This study
**Cellulose synthase** **_Fwd**	GGTCTCCATGTCCCTCTACG	RT-qPCR	This study
**Cellulose synthase** **_Rev**	CAGTTGGGGATCTTGAAGCG	RT-qPCR	This study
**TBPF_Fwd**	GCCGGACAAGAAGCGAAGGAAG	RT-qPCR	This study
**TBPF_Rev**	GTCGGTGAAGTAGACGCGGAAG	RT-qPCR	This study
**Ac18S_Fwd**	TCCAATTTTCTGCCACCGAA	RT-qPCR	[28]
**Ac18S_Rev**	ATCATTACCCTAGTCCTCGC	RT-qPCR	[28]
**ACA1_384820_Fwd_NdeI**	TTTTTTCATATGGACTGCACAACAGAC	Cloning	This study
**ACA1_384820_Rev_XhoI**	TTTTTTCTCGAGTCAACTGGGTGCCGC	Cloning	This study

‘Fwd’ for forward primer and ‘Rev’ for reverse primer. The restriction site added was underlined.

## Supplementary Materials

The following are available online at https://www.mdpi.com/2076-0817/9/5/321/s1.
